# Impact of pulse duration on Ho:YAG laser lithotripsy: fragmentation and dusting performance

**DOI:** 10.1007/s00345-014-1429-8

**Published:** 2014-11-04

**Authors:** Markus J. Bader, Thomas Pongratz, Wael Khoder, Christian G. Stief, Thomas Herrmann, Udo Nagele, Ronald Sroka

**Affiliations:** 1 Department of Urology, Kreisklinik Ebersberg Gemeinnützige GmbH, Ebersberg, Germany; 2 Laser Research Laboratory (LFL) in LIFE-Centre, Hospital of University Munich, Feodor-Lynen-Str. 19, 81377 Munich, Germany; 3 Department of Urology, Hospital of University Munich, Hanover, Germany; 4 Department of Urology and Urooncology, Hanover Medical School (MHH), Munich, Germany; 5 Department of Urology and Andrology, Tiroler Landeskrankenanstalten GmbH, Hall in Tirol, Austria

**Keywords:** Ho:YAG laser, Laser lithotripsy, Laser pulse duration, Fragmentation, Dusting

## Abstract

**Objectives:**

In vitro investigations of Ho:YAG laser-induced stone fragmentation were performed to identify potential impacts of different pulse durations on stone fragmentation characteristics.

**Materials and methods:**

A Ho:YAG laser system (Swiss LaserClast, EMS S.A., Nyon, Switzerland) with selectable long or short pulse mode was tested with regard to its fragmentation and laser hardware compatibility properties. The pulse duration is depending on the specific laser parameters. Fragmentation tests (hand-held, hands-free, single-pulse-induced crater) on artificial BEGO stones were performed under reproducible experimental conditions (fibre sizes: 365 and 200 µm; laser settings: 10 W through combinations of 0.5, 1, 2 J/pulse and 20, 10, 5 Hz, respectively).

**Results:**

Differences in fragmentation rates between the two pulse duration regimes were detected with statistical significance for defined settings. Hand-held and motivated Ho:YAG laser-assisted fragmentation of BEGO stones showed no significant difference between short pulse mode and long pulse mode, neither in fragmentation rates nor in number of fragments and fragment sizes. Similarly, the results of the hands-free fragmentation tests (with and without anti-repulsion device) showed no statistical differences between long pulse and short pulse modes.

**Conclusion:**

The study showed that fragmentation rates for long and short pulse durations at identical power settings remain at a comparable level. Longer holmium laser pulse duration reduces stone pushback. Therefore, longer laser pulses may result in better clinical outcome of laser lithotripsy and more convenient handling during clinical use without compromising fragmentation effectiveness.

## Introduction

The treatment of urinary stones throughout the whole urinary tract via an endoscopic approach has gained widespread acceptance due to technical advancements in endoscope and lithotripter techniques [1–4]. The pulsed holmium:YAG (Ho:YAG) laser has become the preferred lithotripter device [5–7]. One major advantage of this energy source is that laser energy can be delivered through flexible optical fibres that can be advanced through flexible and rigid endoscopes. The Ho:YAG-laser is capable to fragment stones of any composition and hardness; consequently a high stone free rate is achievable. A subject of highest importance in Ho:YAG-laser research is the reduction of the mean stone fragment size in order to improve the discharge of fragments from the urinary tract and to increase treatment success. This process is called ‘stone dusting’.

Currently, the term ‘stone dusting’ stands for laser settings with low energy per pulse and a high pulse repetition rate. Today, this treatment approach is mainly the domain of multi-cavity high-power Ho:YAG laser systems which are able to operate at pulse frequencies of more than 40 Hz.

Sea et al. [8] found that fragmentation did not increase in any consistent fashion when frequency was increased and energy per pulse was held constant. That finding was also confirmed by Chawla et al. [9] who showed that fragmentation rates increase always with pulse energy but not consistently with pulse frequency.

Low repetition rate and high energy per pulse settings produce always higher fragmentation rates compared to high repetition rate and low power setting [10]. Thus, it could be assumed that a Ho:YAG laser system which would allow for working at higher pulse energies to produce increased fragmentation rates while keeping stone migration at a minimum would be an optimal laser lithotripsy system.

Recent medical approved holmium laser developments enable the surgeon to choose between shorter and longer pulse durations in combination with fixed energy per pulse and pulse repetition rate settings. The pulse durations with these recently developed lasers are between 150 and 1,500 µs (flash lamp activation time) compared to pulse durations of 350 and 700 µs described in the earlier studies [11–13]. The aim of this in vitro study was to perform objective and reproducible experiments to determine differences in stone fragmentation between shorter (300–700 µs) and longer (600–1,500 µs) laser pulse duration regimes.

## Materials and methods

Experiments were performed under reproducible conditions to investigate whether relevant effects of pulse duration on lithotripsy could be detected.

To investigate the effects of Ho:YAG laser-induced fragmentation in relation to the pulse duration, hand-held as well as hands-free fragmentation experiments were performed.

### Laser system

The experiments were performed using the 2.1 μm emitting Ho:YAG-laser Swiss LaserClast^®^ (EMS Electro Medical Systems S.A., Nyon, Switzerland) with a maximum power output of 20 W for fibres with core diameters larger than 300 μm. Initial testing of a prototype of the EMS Swiss LaserClast^®^ requested a redesign of the optical system to establish emission stability when working in long pulse mode for an extended period of time. These changes resulted in an improved Ho:YAG laser serial device providing energy emission modes with long as well as short pulse duration in each power setting with high output reproducibility and stability.

The CE approved Swiss LaserClast^®^ Ho:YAG laser allows for energy/pulse settings between 0.5 and 3.5 J/pulse and pulse repetition rates between 3 and 20 Hz. Fibres with core diameters of 200, 272, 365, and 550 μm are available for operation with the system. The pulse duration varies between 300 and 1,500 μs depending on the selected long or short pulse duration range as well as on the energy/pulse and pulse repetition rate setting. According to the system specification, the long pulse/short pulse duration ratio (LP/SP) is at a factor of 1.5–2.5 and depends on the laser parameter used. From the broad variety of selectable laser parameters, the experiments were mainly performed at 10 W-laser power output which is the most common clinical power setting for Ho:YAG laser lithotripsy [8]. Two different bare fibre sizes of 200 and 365 μm were used.

### Hand-held fragmentation

**Fig. 1:**
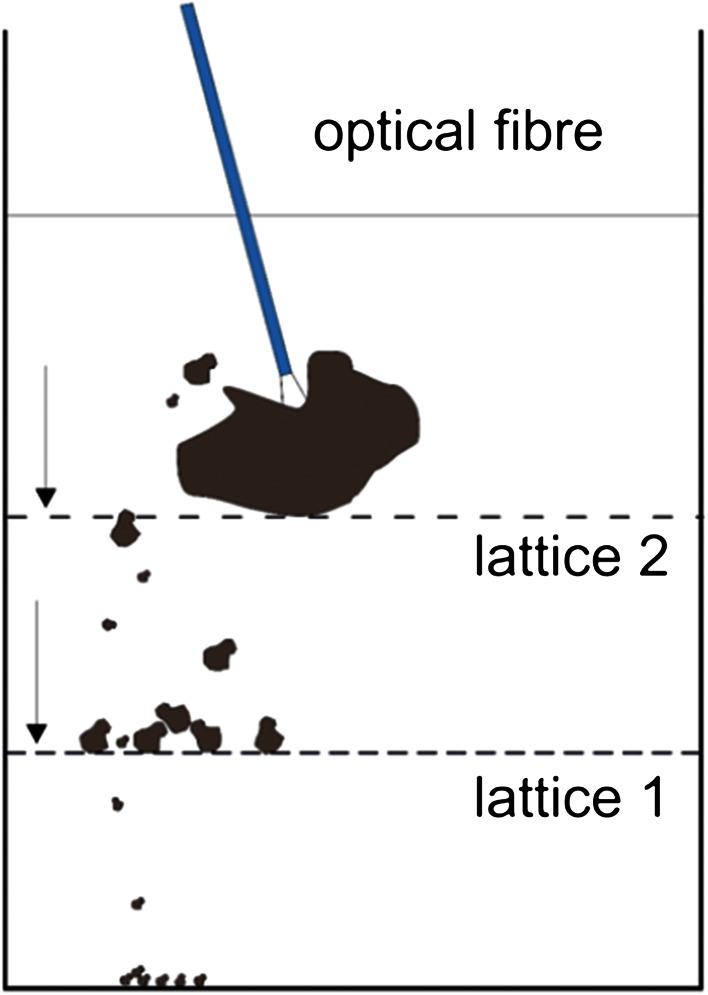
Experimental set-up for hand-held fragmentation testing to separate fragments of different sizes by different lattice mesh sizes. Fragments of <3 mm passed lattice 1 and were retained by lattice 2 which allowed only passage of fragments smaller than 1 mm in size

Hand-held fragmentation tests were performed on cubicle BEGO stone phantoms of defined size (7 × 7 × 7 mm^3^) and hardness (mixing ratio: water/BEGO = 4/15). One highly motivated practitioner performed the fragmentation testing in a standard experimental set-up [14, 15] using a lattice with a mesh size of 3 mm as first layer and a lattice with a 1-mm mesh as second layer as shown in Fig. 1. For that experiment, freshly cleaved 365 μm bare fibres were exclusively used. The fibre tip was brought in nearby contact to the BEGO stone phantom. The output power of the Ho:YAG laser was fixed to 10 W thus allowing for a number of parameter combinations like energy/pulse 0.5, 1.0, and 2.0 J/pulse and repetition rates of 20, 10, and 5 Hz. For each parameter combination at 10-W output power, the specific SP and LP mode was tested with *n* = 10 experiments. Tests were conducted at continuous firing of the laser for 5 min, thus applying total laser energy of 3 kJ.

**Fig. 2:**
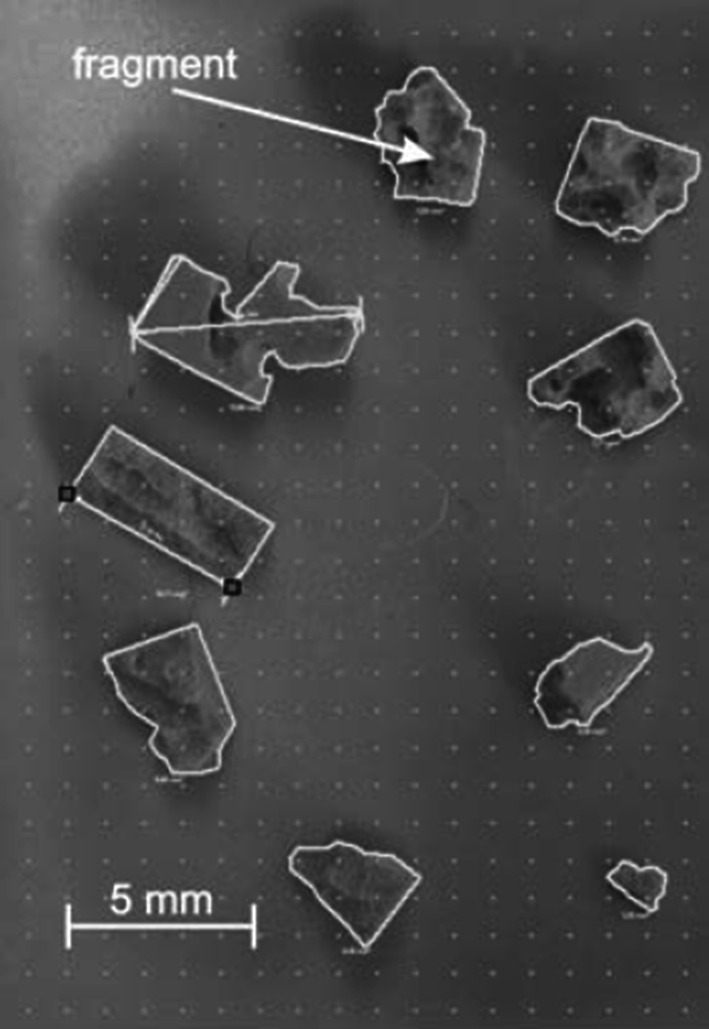
Graphical evaluation of fragment size area for fragments larger than 1 mm by tracing a *polygonal line* to determine the size of the area of each single fragment thus calculating its size (Datinf Measure, Datinf GmbH, Tuebingen, Germany). The quadrat served as reference with 5 mm edge length

Evaluation included weighing of the stone samples prior to laser light application and of the residual fragments remaining on the second lattice layer after each test runs to calculate the ratio of fragments of <1 mm in size (dust) by weight subtraction. Laser activation time and number of laser pulses applied were documented. In order to get information about the size and size distribution of the fragments, for each stone phantom, photographs of the collected fragments from the second lattice were taken. On the base of the photographs, the shape of each fragment was traced by a polygonal line to determine the size of the area of each single fragment (Datinf Measure, Datinf GmbH, Tuebingen, Germany) as shown in Fig. 2. The distribution of the fragment area over area categories (1–3 mm^2^/3–6 mm^2^/6–9 mm^2^ and >9 mm^2^) was evaluated for long and short pulse mode in order to determine a trend difference in fragment size production between the two pulse modes.

### Hands-free fragmentation

**Fig. 3:**
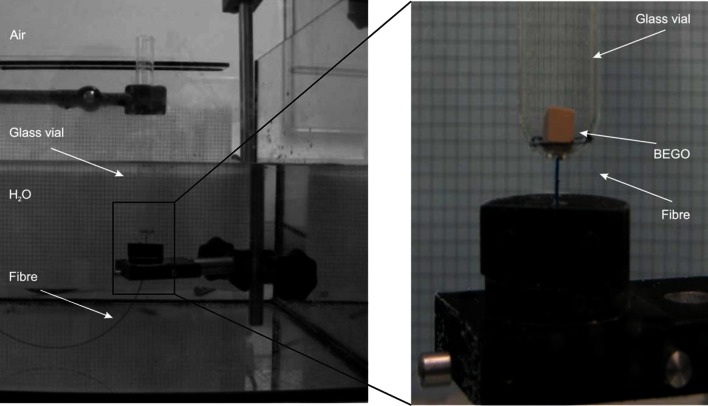
Hands-free fragmentation testing set-up showing the positioning of the fibre in the hole of the bottom of the vial and of the BEGO stone above the fibre

To exclude eventual bias on behalf of the experimenter, fragmentation in a hands-free experimental set-up was additionally tested. The set-up, shown in Fig. 3, consisted of a glass vial with a hole (*d* = 2.5 mm) drilled into the bottom in which the fibre (OD < 1 mm) was positioned by means of holders. Fibre tip and vial bottom were aligned. As the bottom of the vial is of spherical shape, there was a small distance between BEGO stone and the fibre tip when starting each test. The output power of the laser was fixed to 10 W, thus allowing different energy/pulse (0.5, 1.0, and 2.0 J/pulse) and pulse repetition rate (20, 10, and 5 Hz) combinations when operating the 365 μm fibre and 10 and 5 Hz at energy/pulse values of 1.0 and 2.0 J/pulse when using the 200 µm fibre. Each laser setting was tested in its specific SP and LP mode with *n* = 10 experiments per group. Under that setting regime, a total energy of 2 kJ in permanent application mode was applied to the stone sample. Additionally, a round platelet was positioned above the stone in the vial to simulate the effect of an anti- retropulsion device on fragmentation outcome (pushback stopper).

Debris leaved the tube through the space between fibre tip and glass ring. Evaluation included weighing of the stone samples prelaser application and of fragments remaining in the tube after 2 kJ laser energy to calculate the dust by subtraction. Laser activation time and applied number of laser pulses were documented.

### Statistical evaluation

Statistical evaluation included the calculation of mean and standard deviation and its graphical presentation. The one-way ANOVA test including the Bonferroni *t* test was used to calculate statistical significance with *p* < 0.05 as significance level (SigmaPlot 11.0, Systat Software GmbH, Erkrath, Germany).

## Results

### Hand-held fragmentation

**Fig. 4:**
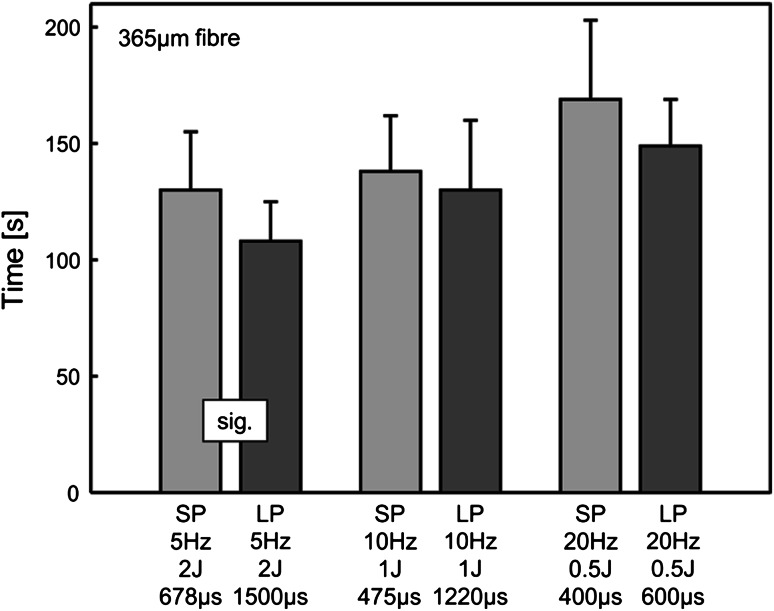
The comparison of the time for destruction of BEGO stone phantoms between SP and LP mode in a hand-held fragmentation test shows no significant difference between both pulse modes, but at 2 J/pulse and 5 Hz

As shown in Fig. 4, the time to destroy a BEGO stone is not significantly different between SP and LP mode. This applies in the fragmentation experiments for the hand-held as well as for the hands-free testing. According to the evaluated quantities like number of laser pulses used, weight of residual fragments on lattice 2 and the weight of the calculated ‘dust’, use or non-use of pushback stopper, fragmentation in LP mode showed a slight tendency to be more effective than in SP mode. This tendency could, however, not be shown for all settings. Thus, no significant difference between LP mode and SP mode in fragmentation could be shown. Without regard to the pulse duration, both modes, however, showed an improved fragmentation performance at higher energy per pulse, in terms of reduced time for destruction, less number of pulse necessary, reduced weight of residual fragments on lattice 2, and finally in increased calculated weight of ‘dust’.

The comparative evaluation of the area of fragments larger than 1 mm is shown in Fig. 2 and listed in Table 1 showed significant differences between the SP and LP group neither for the chosen fragment size ranges nor for the number of fragments. The difference of the median size over all fragments was around 15 % and was lower for long pulse mode. Fragmentation is partly a stochastic process.

**Table 1:** Analysis of fragment sizes larger than 1 mm for long and short pulse mode at 1 J/10 Hz applied over 5 min (*n* = 10 per pulse mode)

Category (mm^2^)	Pulse mode LP–SP	No of fragments	Mean fragment size (mm^2^)	SD fragment size (mm^2^)	Median fragment size (mm^2^)	*p* value
1–3	LP	23	2.1	0.7	2.1	0.08
	SP	21	2.0	0.5	2.1	
3–6	LP	27	4.7	0.9	4.9	0.25
	SP	20	4.8	1.0	4.5	
6–9	LP	36	7.7	0.9	7.5	0.32
	SP	27	7.9	1.0	7.9	
>9	LP	26	7.7	2.3	10.7	0.32
	SP	39	7.9	1.0	7.9	
1–9	LP	112	6.7	3.6	6.8	0.13
	SP	107	7.1	3.9	7.9	

### Hands-free fragmentation

**Fig. 5:**
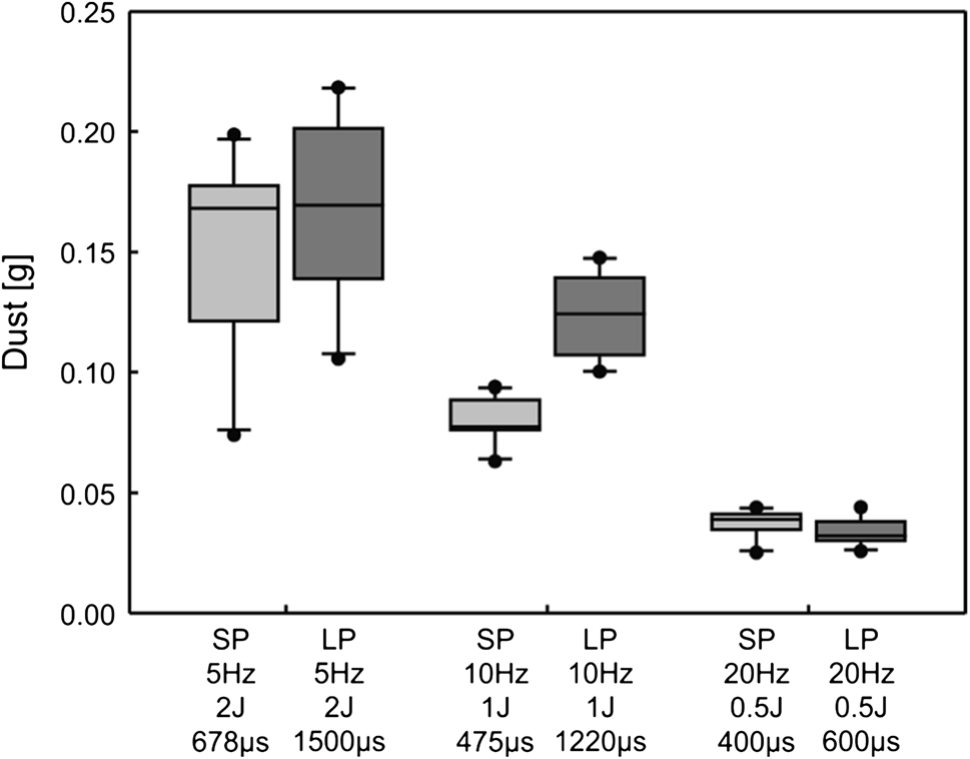
’Dust’ generation during BEGO stone fragmentation in the hands-free experiment (365-µm fibre, total applied energy 2 kJ, power output 10 W, *n* = 10). Comparison between LP and SP mode showed no significant difference except at the laser parameter 1 J and 10 Hz

The hands-free fragmentation tests showed similar dependencies as in the hand-held experiments. The results of ‘dust’ generation during BEGO stone fragmentation using the 365-µm fibre after permanent laser pulse application up to 2 kJ is shown in Fig. 5. Obviously, the amount of ‘dust’ could be significantly increased by heightening the amount of energy per pulse. Comparing the pulse duration modes while keeping the other laser parameter constant, only a significant difference was obtained for a 1 J and 10 Hz setting with the 365 µm fibre.

Other settings performed with the 200-µm fibre did not show a significant difference. Additional hands-free experiments with elimination of repulsion by using a pushback stopper did not change that result. Generally, the long pulse mode showed a slight but not significant tendency to be more effective than the SP mode.

Hence, no significant difference between LP mode and SP mode fragmentation could be demonstrated. However, in both modes, higher pulse energy settings yielded in higher fragmentation.

## Discussion

In this study, in vitro fragmentation experiments were performed by means of an optimized Ho:YAG laser with reproducible long pulse and short pulse modes in order to investigate pulse duration related differences in fragmentation. Both, hand-held as well as hands-free experimental set-ups were created. While a hands-free fragmentation set-up eliminates bias, it also eliminates a potentially positive impact by the motivation of the experimenter conducting the experiment. Thus, on the one hand, the results may become more objective, but on the other hand the hand-guided experiment may be more related to the clinical situation in which the clinician often uses tricky manoeuvres to obtain a completely stone-free outcome. To investigate the influence of endourological stone fixation devices, a stone pushback stopper was added to the hands-free experiment as a second variant. Each experimental setting showed likewise its limitations.

The performed experiments showed that the reported subjective impressions of surgeons during Ho:YAG lithotripsy procedures that longer pulses create smaller fragments than shorter pulses can only be reproduced partly for certain settings by standardized experiments performed in this study. Thus, the perceived clinical difference on the fragment sizes is therefore either correlated with other effects or the difference is too small for a detectable statistical threshold in the experiments. This study represents the results induced by stochastic effects. Beyond that, single pulse effects should be taken into account to achieve a broader view on the underlying processes.

The findings that repulsion depends on pulse duration combined with the results of the current study that fragmentation rates are not reduced at longer pulse durations suggests that improvements in endoscopic Ho:YAG laser lithotripsy are possible by using longer pulses [12, 13]. The short pulse mode may have a clinical advantage when treating immobile urinary stone where repulsion can be neglected and thus a faster coarse fragmentation of stones can be achieved.

The pulse duration with most Ho:YAG lasers is mainly driven by the activating discharge tube and cannot be adjusted by the operator. Some investigations on the effect of variable pulse duration of the Ho:YAG laser pulses were performed [11–13]. Pulse durations investigated in those studies were typically 350 µs (short pulse) and 700 µs (long pulse). It was found that using a holmium laser with longer pulse duration resulted in less object migration after one shock and more energy delivery to an object during repetitive shocks prior to significant displacement. In that study, however, differences in stone migration between the two adjustable pulse durations were only visible at higher pulse energies of >1.6 J/pulse, which suggests that the migration measurement model employed in that study was not very sensitive [13]. The clinically relevant pulse energy range for the treatment of ureteral stones is rather in the lower energy range between 0.8 and 1.2 J than in the higher energy range. Thus, the detection of laser pulse duration related differences in stone migration would be of particular interest for that lower energy range. Furthermore, it was reported that fragmentation effectiveness increases with pulse energy and at shorter pulse duration and recommended those settings for impacted and immobile stones [11].

Currently, several commercially available Ho:YAG-laser systems offer long and short pulse duration settings. According to the manufacturer specifications, however, there are substantial differences concerning the maximum pulse durations, ranging from 700 to 1,500 µs. The actual pulse duration furthermore depends on the adjusted energy per pulse. Emission of long duration pulses in a stable and repetitive manner can be challenging for Ho:YAG laser systems and needs to be confirmed by related verification bench testing for each laser system operating at extended pulse durations.

A Ho:YAG laser system that would allow working at higher pulse energies to produce higher fragmentation rates while keeping stone migration at a minimum would be an ‘ideal’ system design. As for the size of fragments, a standard concerning the size of ‘dusted’ fragments is not yet defined. Low pulse energies produce small debris and less repulsion, but a strategy of low 0.2 J power and high frequency may be not efficient for hard stones [8]. A consensus on how to define stone-free status and success for endoscopic stone treatment is under discussion.

A recently performed PCNL–RCT study [16] reported about an expert consensus via an online Delphi process on PCNL treatment success and stone-free outcome definition. The treatment success definition of a clinically insignificant residual fragment (CIRF) was for 56.8 % of experts defined as fragments of <4 mm and for 31.8 % as <2 mm [16]. However, for fragments of <4 mm, there is a 20 % probability that a future stone event can be expected [17]. Fragments of <2 mm may be considered as a treatment objective if complete stone clearance during the intervention cannot be reached [17]. The ability of Ho:YAG laser energy to produce such small fragments is dependent on laser parameters and settings (energy per pulse, pulse repetition rate, and pulse duration).

Turning the discussion back to the clinical impact of using different pulse durations and their induced effects, it must be noted that stone particle size applicable for the term ‘stone dusting’ has not been established. There is, however, a growing need to define this term. From the physics point of view, ‘dusting’ occurs if disintegration results in particles of sizes in the microscopic scale which may sediment in solution, remain as powder on the bottom of a vial after drying or have to be separated by filtration with filters of microscopic pores. In clinical reality, fragments resulting from disintegration are particles of sizes that are visible through endoscopes. During experimental as well as clinical Ho:YAG laser application, fragments of <1 mm can be observed. In a clinical setting, the fragment size could be easily estimated in relation to the fibre core diameter (e.g. 200 or 365 µm). A recent study confirmed that urologists can estimate fragment size versus fibre diameter with sufficient accuracy [18]. This suggests that targeting a fragment size close to fibre diameter may be a suitable treatment objective to obtain maximum stone clearance.

## Conclusion

This in vitro stone fragmentation study showed that Ho:YAG laser pulse application with pulses duration between 700 and 1,500 µs results in fragmentation capabilities which is comparable to the fragmentation capabilities induced by Ho:YAG laser pulse duration of 150 to 700 µs while having the same laser parameter settings. A critical discussion to define the term ‘dust’ in relation to fragmentation and stone-free rate should be initiated.
